# Post-Exposure Vaccination Improves Gammaherpesvirus Neutralization

**DOI:** 10.1371/journal.pone.0000899

**Published:** 2007-09-19

**Authors:** Laurent Gillet, Janet S. May, Philip G. Stevenson

**Affiliations:** Division of Virology, Department of Pathology, University of Cambridge, Addenbrookes Hospital, Cambridge, United Kingdom; University of California at San Francisco, United States of America

## Abstract

Herpesvirus carriers transmit infection despite making virus-specific antibodies. Thus, their antibody responses are not necessarily optimal. An important question for infection control is whether vaccinating carriers might improve virus neutralization. The antibody response to murine gamma-herpesvirus-68 (MHV-68) blocks cell binding, but fails to block and even enhances an IgG Fc receptor-dependent infection of myeloid cells. Viral membrane fusion therefore remains intact. Although gH/gL-specific monoclonal antibodies can block infection at a post-binding step close to membrane fusion, gH/gL is a relatively minor antibody target in virus carriers. We show here that gH/gL-specific antibodies can block both Fc receptor-independent and Fc receptor-dependent infections, and that vaccinating virus carriers with a gH/gL fusion protein improves their capacity for virus neutralization both *in vitro* and *in vivo*. This approach has the potential to reduce herpesvirus transmission.

## Introduction

Persistent viruses have evolved to co-exist with established host immunity. Herpesviruses are among the most successful, and provide an archetype for many of the immune evasion mechanisms that underlie effective persistence [1]–[3]. In contrast to the plethora of information about T cell evasion, relatively little is known about how herpesviruses evade neutralization by antibody. They must do so, since they continue to transmit infection despite eliciting virus-specific antibodies; by contrast, pre-existing antibodies generally block the transmission of non-persistent viruses. Epidemiological evidence would suggest that herpesvirus antibody evasion is efficient enough even to avoid much selection of viral antigenic variants [4].

It is important when considering antibody evasion to distinguish Fab-dependent neutralization from Fc-dependent antibody functions such as cytotoxicity and opsonization. Herpesviruses transmit between hosts as cell-free virions. Here, antibody evasion must be an evasion of neutralization. In contrast, herpesviruses mainly spread within their hosts via cell/cell contacts [5], [6]. These limit virion exposure to antibody [7], so antibody-dependent cytotoxicity is probably a more important host defence than neutralization [8], [9]. Other Fc receptor (FcR)-dependent effector mechanisms may also operate [10], [11]. Alpha-herpesviruses encode FcR homologs [12] that inhibit host FcR-dependent functions [13]. This reflects that their latency in terminally differentiated neurons makes host colonization highly dependent on lytic spread. In contrast, gamma-herpesviruses colonize their hosts mainly by latency-associated lymphoproliferation [14]–[16]. This may explain why they do not encode FcRs. Yet gamma-herpesvirus must still evade neutralization. A blockade of cell binding by immune serum neutralizes murine gamma-herpesvirus-68 (MHV-68) for infection of FcR^−^ cells, but not FcR^+^ cells [17]. Thus, opsonization can rescue the infectivity of antibody-coated virions. This implies that virion membrane fusion still operates, since it is an essential step in infection by any route. FcR-dependent infection is also described for beta-herpesviruses [18], [19]. Although MHV-68 productively infects FcR^+^ cells, latency usually pre-dominates and virus production is therefore more protracted than in epithelial cells [17], [20]. Thus, with opsonization the likely effects of antibody on transmission and cell/cell spread diverge. By diverting virions into myeloid cells, antibody should damp down MHV-68 lytic infection even when it fails to achieve neutralization. It is the need for transmission rather than the capacity to cause disease that drives viral evolution. A disease readout is therefore not the best way to understand gamma-herpesvirus antibody evasion.

The MHV-68 glycoprotein-specific antibody response predominantly targets gp150, and gp150-specific antibodies account for most of the FcR-dependent infection that is driven by immune sera [21]. Monoclonal antibodies (mAbs) directed against gH/gL block infection at a post-binding step close to membrane fusion [22]. However, gH/gL appears to be poorly immunogenic, as gH/gL-specific antibodies are only a minor component of the total response [21]. Viral membrane fusion remains obligatory whatever the route of infection. So if gH/gL-specific antibodies can also block FcR-dependent infection, a weak gH/gL-specific response might be crucial to viral evasion of neutralization, and inducing stronger gH/gL-specific immunity might be a means of reducing viral spread. The steady state CD8^+^ T cell response of MHV-68 carrier mice is not fixed, but can be altered by post-exposure vaccination [23]. Such an approach also has the potential to alter antibody responses. We have tested here whether the presentation of gH/gL alone, without its usual accompaniment of more immunogenic MHV-68 virion glycoproteins, can shift the antibody response of virus carriers towards better neutralization.

## Results

### gH/gL-specific antibodies inhibit FcR-dependent MHV-68 infection

**Figure 1:**
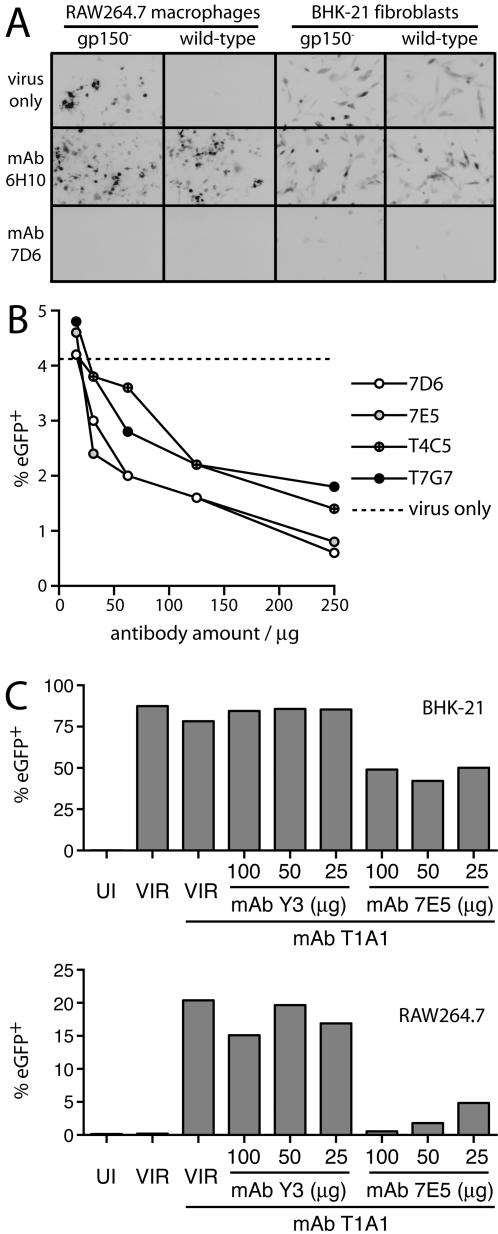
Global inhibition of MHV-68 infection by gH/gL-specific mAbs. A. Wild-type or gp150-deficient (gp150^−^) eGFP-expressing MHV-68 virions were incubated (1 mg mAb/10^4^ p.f.u.) with mAb 6H10 (anti-gp70, IgG2a, non-neutralizing) or mAb 7D6 (anti-gH/gL, IgG2a, neutralizing), or with no antibody (virus only), then added to RAW264.7 macrophages (1 p.f.u./cell) or BHK-21 fibroblasts (0.1 p.f.u./cell). 18 h later, infected cells were identified by viral eGFP expression, and appear dark in this image. The data are from 1 of 5 equivalent experiments. B. Wild-type eGFP-expressing MHV-68 virions (10^5^ p.f.u.) were incubated (2 h, 37°C) with 1 of 4 different gH/gL-specific mAbs or without antibody (virus only). The virus/antibody mixtures were then added to RAW264.7 cells (1 p.f.u./cell). 18 h later, infected cells were enumerated by flow cytometry of viral eGFP expression. The dashed line shows the level of infection with virus alone. The data are from 1 of 3 equivalent experiments. C. EGFP-expressing MHV-68 was incubated (2 h, 37°C) with mAb T1A1 (20 µg/ml) or not, plus either the neutralizing, gH/gL-specific mAb 7E5 or the anti-H2-K^b^ mAb Y3 as a negative control. The virus/antibody mixtures were then used to infect BHK-21 fibroblasts (1 p.f.u./cell) or RAW264.7 macrophages (5 p.f.u./cell). Infection was quantitated 18 h later by flow cytometry of viral eGFP expression. UI = uninfected, VIR = virus only. The data are from 1 of 2 equivalent experiments.

We first sought to establish whether gH/gL-specific mAbs could block FcR-dependent infection (Fig. 1). We have shown previously [17] that RAW264.7 macrophages provide a reasonable model of the interaction between antibody-coated virions and FcR^+^ cells, and have validated eGFP expression from the MHV-68 genome as a comparative measure of infection between cell populations [17]. We therefore used this system for *in vitro* analysis. In contrast to the non-neutralizing gp70-specific mAb 6H10, the neutralizing gH/gL-specific mAb 7D6 failed to enhance the infection of RAW264.7 macrophages by wild-type MHV-68 and inhibited their infection by gp150-deficient MHV-68 (Fig. 1A). (Gp150-deficient MHV-68 shows enhanced infection of cells such as macrophages that have low glycosaminoglycan expression [24], [25].) MAb 7D6 also blocked BHK-21 cell infection by both viruses. Baseline RAW264.7 cell infection by wild-type MHV-68 was too low to identify clear reductions by fluorescence microscopy, but an inhibition of infection by various gH/gL-specific mAbs was evident on flow cytometry (Fig. 1B). A gH/gL-specific mAb was also able to reverse the FcR-dependent infection driven by a non-neutralizing gp150-specific mAb (Fig. 1C).

### The gH/gL-specific component of immune serum limits FcR-dependent MHV-68 infection

**Figure 2:**
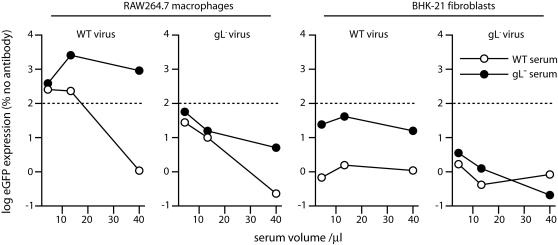
gH/gL-specific antibodies inhibit RAW264.7 macrophage infection by virions exposed to immune serum. Wild-type or gL-deficient (gL^−^) eGFP-expressing virions were incubated with dilutions of immune sera (2 h, 37°C), pooled from 3 mice infected 3 months before with wild-type or gL-deficient MHV-68. The virus/antibody mixtures were then added to RAW264.7 macrophages (3 p.f.u./cell) or BHK-21 fibroblasts (0.3 p.f.u./cell). 18 h later, LPS (350 ng/ml) was added for 6 h to maximize viral eGFP expression [17] and infection was quantitated by flow cytometry of eGFP^+^ cells. Each value is expressed as a percentage of the eGFP expression with virus alone. A log scale is used to encompass the huge range between infection enhancement and neutralization. Virus alone would be 100%, or log = 2 (dashed line). The data are from 1 of 2 equivalent experiments.

Although immune sera mainly promote FcR-dependent MHV-68 infection [17], very high doses of some sera can be inhibitory. To test what gH/gL-specific antibodies normally contribute to the effect of whole serum on FcR-dependent infection, we compared sera from mice infected with wild-type or gL-deficient [26] MHV-68 (Fig. 2). gL-deficient MHV-68 mutants colonize mice much like the wild-type [26], and are just equally immunogenic as measured by ELISA for total MHV-68-specific serum antibody (data not shown). However, they elicit no gH/gL-specific antibodies, because they express no gL-dependent epitopes [26].

Wild-type immune serum neutralized both wild-type and gL-knockout virions for BHK-21 cell infection. gL knockout-immune serum neutralized wild-type MHV-68 relatively poorly, consistent with gH/gL being a major neutralization target [22]. But much more striking was its very strong enhancement of FcR-dependent infection by wild-type virions. In contrast, gL knockout-immune serum inhibited FcR-dependent infection by gL-knockout virions. This may reflect that gH alone is more readily neutralized than gH/gL. Note that gH-specific antibodies are also present in wild-type immune sera [26]. Thus, it appeared that both monoclonal and polyclonal gH/gL-specific antibodies can inhibit FcR-dependent MHV-68 infection. This argued that the level of gH/gL-specific immunity critically determines the fate of antibody-exposed virions.

### Boosting gH/gL-specific immunity reduces IgG FcR-dependent and FcR-independent infections

**Figure 3:**
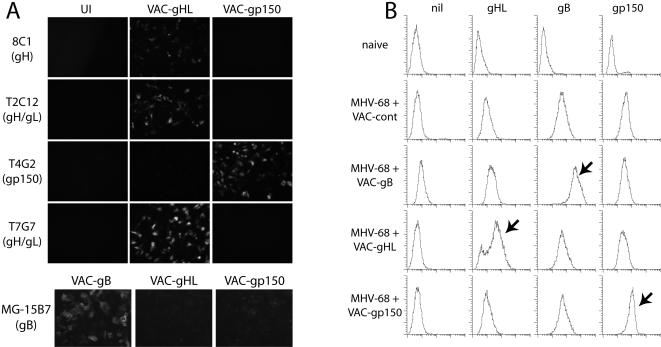
Boosting MHV-68 carrier mice with MHV-68 glycoproteins alters the composition of immune sera. A. Vero cells were left infected (UI) or infected (1 p.f.u./cell, 18 h) with vaccinia virus recombinants as indicated, then fixed, permeabilized and stained for MHV-68 glycoproteins. T2C12 and T7G7 see different gH/gL neutralizing epitopes [22]. The data are from 2 separate experiments. B. MHV-68 carrier mice (3 months post-infection) were infected with vaccinia virus recombinants as shown. The control recombinant (VAC-cont) expresses the murine invariant chain with an ovalbumin-derived epitope. Sera were taken 10 days post-infection, pooled from 5 mice per group, and used to stain MHV-68 glycoprotein-expressing CHO cell lines as indicated. nil = untransfected, gHL = transfected with gH/gL-GPI fusion protein, gB = transfected with gB-GPI, gp150 = transfected with gp150. Naive = serum from naive mice. The arrows indicate strong increases in glycoprotein staining. The data are from 1 of 2 equivalent experiments.

We next tested whether neutralizing antibodies can be boosted by post-exposure vaccination of MHV-68 carrier mice. Our first task was to express a suitable form of gH. Although gH alone can reach the cell surface, it does so in a conformation not recognized by neutralizing mAbs [22]. To ensure that gH would adopt a suitable conformation, we fused it to gL. Since a glycosyl-phosphatidyl-inositol (GPI)-linked form of gL can fold gH in transfected cells [22], gL is probably sited close to the membrane in the mature gH/gL heterodimer. We therefore fused gL to the C-terminus of the gH extracellular domain (gHL), again with a GPI anchor. Cells transfected with this fusion protein were recognized by all our gH/gL-specific neutralizing mAbs (n>30, data not shown). For gene delivery, we transferred this construct into vaccinia virus. Cells infected with vaccinia virus expressing the gHL fusion protein (VAC-gHL) displayed gH/gL neutralization epitopes (Fig. 3A). We also generated a vaccinia virus recombinant (VAC-gB) expressing the gB extracellular domain with a GPI anchor, a construct equivalent to one we have used before to identify gB-specific mAbs [27]. A third vaccinia virus, expressing the N-terminal third of gp150 with a GPI anchor, has been described [21]. Infecting MHV-68 carrier mice with each vaccinia recombinant boosted antibody against the cognate MHV-68 glycoprotein, as evident by flow cytometric staining of glycoprotein-transfected cells with immune sera (Fig. 3B).

**Figure 4:**
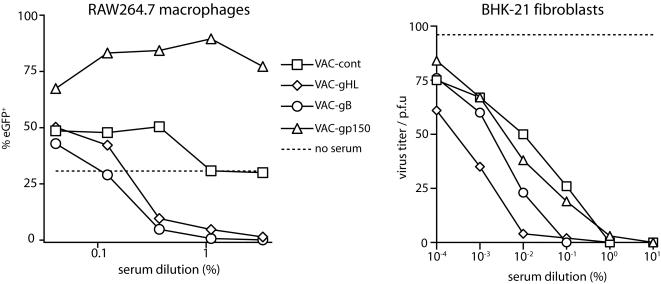
Altered functional impact of sera from MHV-68 carrier mice boosted with individual virion glycoproteins. EGFP-expressing MHV-68 virions were incubated with sera from MHV-68 carrier mice boosted 10 days earlier with vaccinia virus recombinant as shown. The virus/antibody mixtures were then used to infect RAW264.7 macrophages (5 p.f.u./cell) or BHK-21 fibroblasts (100 p.f.u./well). RAW264.7 cell infection was quantitated 18 h later by flow cytometry of viral eGFP expression. BHK-21 cell infection was measured by plaque assay. The dashed lines show infection with virus alone (no serum). The data are from 1 of 2 equivalent experiments.

**Figure 5:**
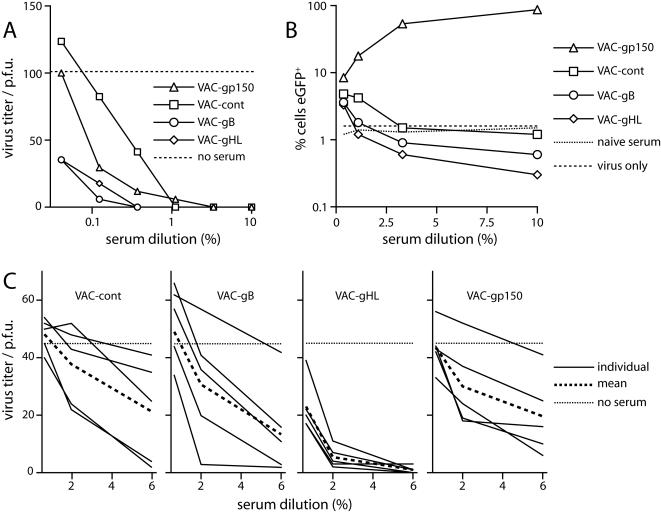
Maintenance of altered neutralization responses after boosting of virus carriers. A. Pooled serum samples (5 mice per group) at 5 months post-boosting of MHV-68 carrier mice with the vaccinia virus recombinant shown, were used to inhibit MHV-68 infection of BHK-21 cells in a plaque reduction assay. The data are from 1 of 3 equivalent experiments. The dashed line shows the titer of the same amount of virus without added antibody. B. The same serum samples as in A were tested for their effect on RAW264.7 cell infection. eGFP-expressing MHV-68 virions were incubated with serum dilutions as shown (2 h, 37°C) then added to RAW264.7 macrophages (3 p.f.u./cell). Infection was assayed 18 h later by flow cytometry of viral eGFP expression. The dashed lines show infection levels for MHV-68 incubated with either no serum or with sera pooled from age-matched naive mice. The data are from 1 of 2 equivalent experiments. C. Sera from individual vaccinia-boosted, MHV-68 carrier mice, again at 5 months post-boosting, were incubated with MHV-68 virions (2 h, 37°C). The infectivity titer of each serum/virus mixture was then determined by plaque assay on BHK-21 fibroblasts. Each solid line shows the result for a serum sample from one mouse. The dashed line shows the mean titer of the group. The dotted line shows the mean titer of 3 virus samples without added immune serum. The data are from 1 of 2 equivalent experiments.

This boosting correlated with a marked change in the impact of immune sera on MHV-68 infectivity: VAC-150 increased RAW264.7 cell infection, consistent with gp150 driving this process [21], but boosting with VAC-gB or VACgHL reduced it (Fig. 4A). Boosting with VAC-gB or VAC-gHL also improved the serum-mediated neutralization of BHK-21 cell infection (Fig. 4B). Five months after boosting, the neutralization titres of sera pooled from VAC-gB-boosted or VAC-gHL-boosted mice had declined somewhat, but they still remained significantly above those of control mice for both fibroblast (Fig. 5A) and RAW264.7 cell (Fig. 5B) infections.

gB-specific neutralizing mAbs are only detectable in a minority of MHV-68-infected mice, possibly because significant neutralization requires IgM isotype antibodies [28] and most MHV-68-specific B cells produce IgG [29], [30]. We therefore also tested individual sera for neutralization (Fig. 5C). The titers of VAC-gB-boosted mice were highly variable. One serum neutralized strongly-and probably dominated the pooled sample-but others overlapped with the controls. In contrast, sera from VAC-gHL-boosted mice all neutralized MHV-68 better than the controls. This was consistent with gH/gL-specific neutralizing mAbs being more reliably recovered from MHV-68 carriers [22]. Thus, gHL was a more uniformly effective post-exposure vaccine for boosting neutralizing antibodies.

### *In vivo* neutralization

**Figure 6:**
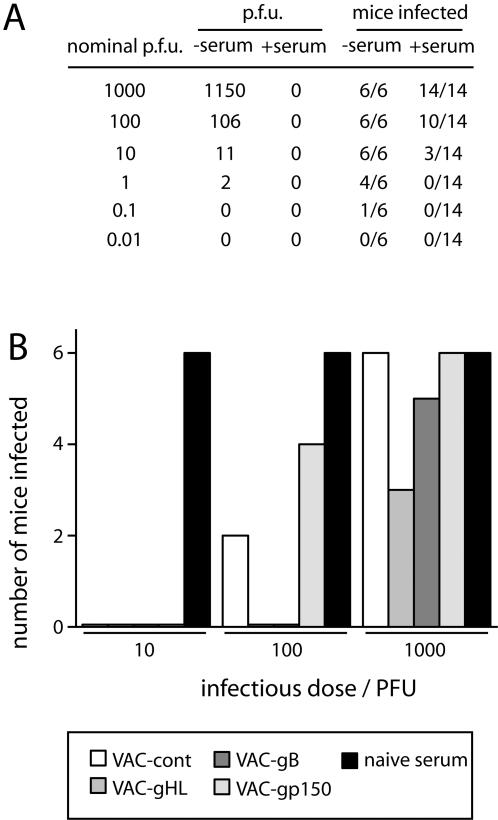
Neutralization of MHV-68 virions for *in vivo* infection. A. Wild-type MHV-68 virions were filtered to remove any aggregates, then diluted as shown (nominal PFU) and incubated or not with 10% serum pooled from mice infected 3 months previously with wild-type MHV-68. Each sample was then titrated on BHK-21 cells (p.f.u.) and used to infect naive mice intranasally (mice infected). Infection of mice was determined by plaque assay of lungs harvested 7 days later, and were scored simply as positive or negative depending on whether any virus was present. The *in vivo* data are pooled from 3 different experiments that all gave consistent results. B. Wild-type MHV-68 virions were incubated with pooled serum samples from vaccinia virus boosted MHV-68 carrier mice as in A, then given intranasally to naive mice (5 µl of serum per mouse with the virus dose shown, using 6 mice per group). Lungs were titered for infectious virus at 7 days post-infection and scored as either infected or not. The data are pooled from 2 equivalent experiments (3 mice per group each).

Extrapolating *in vitro* neutralization to an *in vivo* setting is not straightforward. Herpesvirus entry routes into naive hosts are not well characterized and are probably multiple. We have seen already with IgG Fc receptors that accessory uptake pathways can bypass apparent blocks to infection [17]. Lectin-mediated uptake may do the same [31]. An *in vivo* test of neutralization is therefore desirable to confirm that *in vitro* assays are realistic. One approach to has been to inject mice with congenic [32] or xenogenic [33] immune sera. However, MHV-68 infection is simply too complicated to interpret the effects of such treatments as neutralization without more direct evidence (see Introduction). Indeed, it is unlikely that neutralizing antibodies stop cell/cell viral spread. The major opportunity for neutralizing antibodies to act comes when cell-free virions pass from an infected to a naive host. Therefore one major area of complexity to encompass in neutralization assays is the uptake of antibody-exposed virions at a mucosal surface. With this in mind, we tested the infectivity of virions that had been exposed to immune sera by inoculating them intranasally into naive mice (Fig. 6A). The readout was simply infection or not, based on virus titers in lung homogenates at 7 days post-inoculation.

In the absence of immune serum, *in vivo* infectivity was comparable to *in vitro*: 1 p.f.u. infected 4/6 mice, consistent with a Poisson distribution. Serum-mediated neutralization was much more effective *in vitro* than *in vivo*. Thus, 10% immune serum reduced *in vitro* infectivity at least 1000-fold (1000 p.f.u. was reduced to 0 p.f.u.), but reduced *in vivo* infectivity no more than 100-fold (100 p.f.u. mixed with immune serum still infected 10/14 mice). We then tested *in vivo* neutralization by the boosted sera (Fig. 6B). All MHV-68-immune sera neutralized 10 p.f.u. for intranasal infection, but only the gHL-boosted and gB-boosted sera completely neutralized 100 p.f.u. and only the gHL-boosted sera were able to stop at all (3/6 mice) infection by 1000 p.f.u.. *In vivo* neutralization was therefore improved significantly by post-exposure vaccination.

## Discussion

Immune sera block MHV-68 infection of fibroblasts, but fail to block and even enhance its infection of FcR^+^ cells such as macrophages and dendritic cells. At mucosal surfaces, FcR^+^ and DC-SIGN^+^ [31] dendritic cell processes [34]; mucosal epithelial FcRn [35]; and M cell transcytosis [36] all provide potential uptake routes for antibody-coated virions. Merely blocking cell binding may therefore not suffice to block viral transmission. In contrast, membrane fusion is essential for enveloped virions to infect regardless of the uptake route. It should therefore be a universally effective neutralization target. gH/gL-specific antibodies block MHV-68 infection at a point between cell binding and membrane fusion [22]. We showed here that they can inhibit the infection of both FcR^−^ and FcR^+^ cells, and can therefore potentially block all routes into the naive host.

MHV-68 infection elicits gH/gL-specific antibodies, but quantitative analysis of the glycoprotein-specific antibody reponse indicates that gH/gL is quite poorly immunogenic [21]. gH/gL neutralizing epitopes are lost if gL and gH dissociate [22], and B cells specific for other, more immunogenic virion glycoproteins probably reduce the gH/gL antigen load [21]. The effectiveness of gH/gL mAbs and polyclonal sera in blocking MHV-68 infection argued that poor gH/gL immunogenicity is a crucial factor in allowing antibody-exposed virions to remain infectious *in vivo*. We showed that the steady state antibody response of virus carriers is not fixed, but can be modified by post-exposure vaccination. Subunit vaccination avoided the problem with whole MHV-68 of eliciting antibodies mainly to gp150, which promote FcR-dependent infection [21]. By selectively boosting gH/gL-specific antibody, the balance of immune serum was tipped from promoting FcR-dependent infection to inhibiting it. *In vivo* neutralization-which was notably more difficult for immune sera to achieve than *in vitro* neutralization-was also improved. Immunization with a gH-gL fusion protein therefore has the potential to improve unfavourable antibody responses in herpesvirus carriers, and thereby to reduce their infectivity.

The limitation of this approach is likely to be that herpes virions are intrinsically hard to neutralize. The relatively low efficiency with which gH/gL-specific mAbs neutralize MHV-68 [22] implies that the fusion machinery is quite well protected. Even saturating levels of a MHV-68 gH/gL-specific neutralizing mAb fail to protect against *in vivo* infection with 10 p.f.u. MHV-68 (P.G. Stevenson, unpublished data). Fortunately, polyclonal immune sera generally neutralize more effectively than monoclonal antibodies because different antibody specificities can have synergistic inhibitory effects. It evidently remained feasible for a boosted, polyclonal gH/gL-specific response to block infection *in vivo*. As 1 virion in principle infects no less well than 1000, the neutralization of 100 p.f.u. by gHL-boosted sera implied that the average infectivity of each virion could be reduced to less than 1% of normal for all infection routes. Even if *in vivo* infectivity is not abolished completely, the limited transmissibility of persistent viruses suggests that modest infectivity reductions might suffice to reduce significantly viral prevalence.

One complication of post-exposure vaccination is that several herpesviruses assemble alternative fusion complexes [37]–[39]. The best vaccine would presumably be the predominant fusion complex on shed virions-with EBV that incorporating gp42 [40]. It may prove sufficient to target just the fusion complex associated with the major route into naive hosts, or it may prove necessary to target more than one fusion complex. Another key task is to test post-exposure vaccination in an *in vivo* transmission model. One has not yet been established for MHV-68. Nevertheless, our study of MHV-68 shows that while the difficulty of *in vivo* neutralization should not be under-estimated, it can in principle be achieved by boosting antibodies against appropriate virion glycoproteins.

## Materials and Methods

### Mice

BALB/c mice were purchased from Harlan U.K. Ltd. (Bicester, U.K.), housed in the Cambridge University Department of Pathology (Home Office Project Licence 80/1992). For immunization studies, mice were and infected intranasally with 3×10^4^ PFU MHV-68 when 6–8 weeks old. Vaccinia viruses were given by intraperitoneal injection (3×10^6^ PFU). For infection studies, mice were infected intranasally with virus/antibody mixtures and lungs were removed for plaque assay 7 days later.

### Cells

BHK-21 cells, RAW264.7 cells, CHO and CHO-gB cells [27] cells, L929-gp150 cells [21], NIH-3T3-CRE cells [41] and TK^-^143 cells were grown in Dulbecco's modified Eagle medium (Invitrogen, Paisley, U.K.) supplemented with 2 mM glutamine, 100 U/ml penicillin, 100 µg/ml streptomycin and 10% fetal calf serum (PAA laboratories, Linz, Austria). Where indicated, cells were transfected using Fugene-6 (Roche Diagnostics Ltd., Lewes, U.K.).

### Viruses

MHV-68 was derived from a genomic BAC, which also transcribes eGFP from a human cytomegalovirus IE-1 promoter [42]. gL-deficient [26] and gp150-deficient [24] derivatives have been described. For all *in vivo* infections, the loxP flanked eGFP and BAC sequences were removed by passaging viruses through NIH-3T3-CRE cells. Viruses were grown in BHK-21 cells. Infected cultures were cleared of infected cell debris by low-speed centrifugation (1000×g, 3 min). Virions were then concentrated by high speed centrifugation (38000×g, 90 min). Virus stocks for *in vivo* neutralization assays were also filtered (0.45 µm) to remove any aggregates. Virus titers of stocks, antibody-treated samples and lung homogenates were determined by plaque assay on BHK-21 cells [15]. To make gHL-GPI, the gL coding sequence lacking its stop codon and the first 13 amino acid residues of its signal peptide [22] was amplified by PCR (Phusion DNA polymerase, New England Biolabs, Hitchin, U.K.) with *Not*I-restricted primers and cloned into the *Not*I site between gH and its GPI anchor in pBRAD-gH [22] to make pBRAD-gHL. Residues AFVSLSTC of the predicted gL signal peptide were retained as a linker between gL and gH. CHO cells were transfected with pBRAD-gHL to make CHO-gHL cells. The correct expression of virion gH/gL epitopes by this cell line was confirmed with>30 gH/gL-specific mAbs (data not shown). To make VAC-gHL, the gHL-GPI coding sequence was amplified from pBRAD-gHL using 5′ AvrII-restricted and 3′ HinDIII-restricted primers and cloned into the NheI/HinDIII sites of pMJ601 [43]. To make VAC-gB, the gB-GPI coding sequence was amplified from pBRAD-gB [27], again with 5′ AvrII-restricted and 3′ HinDIII-restricted primers, and cloned into pMJ601 as for VAC-gHL. pMJ-601-gHL-GPI and pMJ601-gB-GPI were each transfected into vaccinia virus WR-infected TK^-^143 cells. Thymidine kinase-deficient recombinants were selected by passage in 25 µg/ml 5′-bromo-2′-deoxyuridine (Sigma Chemical Co, Poole, U.K.) and identified by beta galactosidase expression using X-gal substrate. They were purified to homogeneity by limiting dilution cloning. VAC-gp150 has been described [21].

### Flow cytometry

Cells infected with eGFP-expressing viruses were washed in PBS and analysed directly for green channel fluorescence. For specific staining, cells were incubated with MHV-68 glycoprotein-specific mAbs (1 h, 4°C), washed×2 in PBS, incubated with fluorescein-conjugated rabbit anti-mouse IgG pAb (Dako Cytomation, Ely, U.K.), washed×2, and analysed on a FACS Calibur (Becton-Dickinson, Oxford, U.K.). We used the following mAbs. gH/gL: 7D6, 7E5, T4C5, T7G7, T2C12 (all IgG2a), gp150: T1A1, T4G2 (both IgG2a), gB: MG-15B7 (IgG1), gp70: 6H10 (IgG2a).

### Immunofluorescence

eGFP expression in live cells was visualised directly. For staining with MHV-68-specific mAbs, cells were fixed (4% paraformaldehyde, 30 min), permeabilized (1% Triton-X100, 15 min), blocked with 5% fetal calf serum, then incubated with mAbs (1 h), washed×3 in PBS, incubated with Alexa488-conjugated goat anti-mouse pAb (Invitrogen Corporation, Paisley, U.K.), washed×3 and mounted with Prolong Gold antifade (Invitrogen). Fluorescence was visualized with an Olympus IX70 microscope plus a Retiga 2000R camera line (QImaging)

## References

[1] Tortorella D, Gewurz BE, Furman MH, Schust DJ, Ploegh HL (2000). Viral subversion of the immune system.. Annu Rev Immunol.

[2] Yewdell JW, Hill AB (2002). Viral interference with antigen presentation.. Nat Immunol.

[3] Stevenson PG (2004). Immune evasion by gamma-herpesviruses.. Curr Opin Immunol.

[4] Xu J, Lyons PA, Carter MD, Booth TW, Davis-Poynter NJ (1996). Assessment of antigenicity and genetic variation of glycoprotein B of murine cytomegalovirus.. J Gen Virol.

[5] Dingwell KS, Brunetti CR, Hendricks RL, Tang Q, Tang M (1994). Herpes simplex virus glycoproteins E and I facilitate cell-to-cell spread in vivo and across junctions of cultured cells.. J Virol.

[6] Peeters B, Pol J, Gielkens A, Moormann R (1993). Envelope glycoprotein gp50 of pseudorabies virus is essential for virus entry but is not required for viral spread in mice.. J Virol.

[7] Roth MG, Compans RW (1980). Antibody-resistant spread of vesicular stomatitis virus infection in cell lines of epithelial origin.. J Virol.

[8] Balachandran N, Bacchetti S, Rawls WE (1982). Protection against lethal challenge of BALB/c mice by passive transfer of monoclonal antibodies to five glycoproteins of herpes simplex virus type 2.. Infect Immun.

[9] Kohl S, Loo LS, Schmalstieg FS, Anderson DC (1986). The genetic deficiency of leukocyte surface glycoprotein Mac-1, LFA-1, p150,95 in humans is associated with defective antibody-dependent cellular cytotoxicity in vitro and defective protection against herpes simplex virus infection in vivo.. J Immunol.

[10] Rector JT, Lausch RN, Oakes JE (1982). Use of monoclonal antibodies for analysis of antibody-dependent immunity to ocular herpes simplex virus type 1 infection.. Infect Immun.

[11] Huber VC, Lynch JM, Bucher DJ, Le J, Metzger DW (2001). Fc receptor-mediated phagocytosis makes a significant contribution to clearance of influenza virus infections.. J Immunol.

[12] Johnson DC, Feenstra V (1987). Identification of a novel herpes simplex virus type 1-induced glycoprotein which complexes with gE and binds immunoglobulin.. J Virol.

[13] Nagashunmugam T, Lubinski J, Wang L, Goldstein LT, Weeks BS (1998). In vivo immune evasion mediated by the herpes simplex virus type 1 immunoglobulin G Fc receptor.. J Virol.

[14] Stevenson PG, Belz GT, Castrucci MR, Altman JD, Doherty PC (1999). A gamma-herpesvirus sneaks through a CD8(+) T cell response primed to a lytic-phase epitope.. Proc Natl Acad Sci U S A.

[15] Coleman HM, de Lima B, Morton V, Stevenson PG (2003). Murine gammaherpesvirus 68 lacking thymidine kinase shows severe attenuation of lytic cycle replication in vivo but still establishes latency.. J Virol.

[16] May JS, Coleman HM, Smillie B, Efstathiou S, Stevenson PG (2004). Forced lytic replication impairs host colonization by a latency-deficient mutant of murine gammaherpesvirus-68.. J Gen Virol.

[17] Rosa GT, Gillet L, Smith CM, de Lima BD, Stevenson PG (2007). IgG fc receptors provide an alternative infection route for murine gamma-herpesvirus-68.. PLoS ONE.

[18] Inada T, Chong KT, Mims CA (1985). Enhancing antibodies, macrophages and virulence in mouse cytomegalovirus infection.. J Gen Virol.

[19] Maidji E, McDonagh S, Genbacev O, Tabata T, Pereira L (2006). Maternal antibodies enhance or prevent cytomegalovirus infection in the placenta by neonatal Fc receptor-mediated transcytosis.. Am J Pathol.

[20] Marques S, Efstathiou S, Smith KG, Haury M, Simas JP (2003). Selective gene expression of latent murine gammaherpesvirus 68 in B lymphocytes.. J Virol.

[21] Gillet L, May JS, Colaco S, Stevenson PG (2007). The murine gammaherpesvirus-68 gp150 acts as an immunogenic decoy to limit virion neutralization.. PLoS ONE.

[22] Gill MB, Gillet L, Colaco S, May JS, de Lima BD (2006). Murine gammaherpesvirus-68 glycoprotein H-glycoprotein L complex is a major target for neutralizing monoclonal antibodies.. J Gen Virol.

[23] Belz GT, Stevenson PG, Castrucci MR, Altman JD, Doherty PC (2000). Postexposure vaccination massively increases the prevalence of gamma-herpesvirus-specific CD8+ T cells but confers minimal survival advantage on CD4-deficient mice.. Proc Natl Acad Sci U S A.

[24] de Lima BD, May JS, Stevenson PG (2004). Murine gammaherpesvirus 68 lacking gp150 shows defective virion release but establishes normal latency in vivo.. J Virol.

[25] Gillet L, Adler H, Stevenson PG (2007). Glycosaminoglycan interactions in murine gammaherpesvirus-68 infection.. PLoS ONE.

[26] Gillet L, May JS, Colaco S, Stevenson PG (2007). Glycoprotein L disruption reveals 2 functional forms of the murine gammaherpesvirus-68 glycoprotein H.. J Virol.

[27] Lopes FB, Colaco S, May JS, Stevenson PG (2004). Characterization of the MHV-68 glycoprotein B.. J Virol.

[28] Gillet L, Gill MB, Colaco S, Smith CM, Stevenson PG (2006). Murine gammaherpesvirus-68 glycoprotein B presents a difficult neutralization target to monoclonal antibodies derived from infected mice.. J Gen Virol.

[29] Stevenson PG, Doherty PC (1998). Kinetic analysis of the specific host response to a murine gammaherpesvirus.. J Virol.

[30] Sangster MY, Topham DJ, D'Costa S, Cardin RD, Marion TN, Myers LK, Doherty PC (2000). Analysis of the virus-specific and nonspecific B cell response to a persistent B-lymphotropic gammaherpesvirus.. J Immunol.

[31] van Montfort T, Nabatov AA, Geijtenbeek TB, Pollakis G, Paxton WA (2007). Efficient capture of antibody neutralized HIV-1 by cells expressing DC-SIGN and transfer to CD4+ T lymphocytes.. J Immunol.

[32] Kim IJ, Flano E, Woodland DL, Blackman MA (2002). Antibody-mediated control of persistent gamma-herpesvirus infection.. J Immunol.

[33] Gangappa S, Kapadia SB, Speck SH, Virgin HW (2002). Antibody to a lytic cycle viral protein decreases gammaherpesvirus latency in B-cell-deficient mice.. J Virol.

[34] Niess JH, Brand S, Gu X, Landsman L, Jung S, McCormick BA, Vyas JM, Boes M, Ploegh HL, Fox JG, Littman DR, Reinecker HC (2005). CX3CR1-mediated dendritic cell access to the intestinal lumen and bacterial clearance.. Science.

[35] Bitonti AJ, Dumont JA, Low SC, Peters RT, Kropp KE, Palombella VJ, Stattel JM, Lu Y, Tan CA, Song JJ, Garcia AM, Simister NE, Spiekermann GM, Lencer WI, Blumberg RS (2004). Pulmonary delivery of an erythropoietin Fc fusion protein in non-human primates through an immunoglobulin transport pathway.. Proc Natl Acad Sci U S A.

[36] Kraehenbuhl JP, Neutra MR (2000). Epithelial M cells: differentiation and function.. Annu Rev Cell Dev Biol.

[37] Wang X, Kenyon WJ, Li Q, Mullberg J, Hutt-Fletcher LM (1998). Epstein-Barr virus uses different complexes of glycoproteins gH and gL to infect B lymphocytes and epithelial cells.. J Virol.

[38] Wang D, Shenk T (2005). Human cytomegalovirus virion protein complex required for epithelial and endothelial cell tropism.. Proc Natl Acad Sci USA.

[39] Mori Y, Akkapaiboon P, Yonemoto S, Koike M, Takemoto M, Sadaoka T, Sasamoto Y, Konishi S, Uchiyama Y, Yamanishi K (2004). Discovery of a second form of tripartite complex containing gH-gL of human herpesvirus 6 and observations on CD46.. J Virol.

[40] Jiang R, Scott RS, Hutt-Fletcher LM (2006). Epstein-Barr virus shed in saliva is high in B-cell-tropic glycoprotein gp42.. J Virol.

[41] Stevenson PG, May JS, Smith XG, Marques S, Adler H, Koszinowski UH, Simas JP, Efstathiou S (2002). K3-mediated evasion of CD8(+) T cells aids amplification of a latent gamma-herpesvirus.. Nat Immunol.

[42] Adler H, Messerle M, Wagner M, Koszinowski UH (2000). Cloning and mutagenesis of the murine gammaherpesvirus 68 genome as an infectious bacterial artificial chromosome.. J Virol.

[43] Davison AJ, Moss B (1990). New vaccinia virus recombination plasmids incorporating a synthetic late promoter for high level expression of foreign proteins.. Nucleic Acids Res.

